# Reply to Li, A.-H.; Chiu, Y.-L. Drug–Drug Interactions, Medication Adherence, and Stroke Should Be Considered When Approaching the Impact of Acid Suppression Therapy on Chronic Kidney Disease Patients. Comment on “Chen et al. Impact of Acid Suppression Therapy on Renal and Survival Outcomes in Patients with Chronic Kidney Disease: A Taiwanese Nationwide Cohort Study. *J. Clin. Med.* 2022, *11*, 5612”

**DOI:** 10.3390/jcm12010171

**Published:** 2022-12-26

**Authors:** Yi-Chun Chen, Ben-Hui Yu, Wen-Yen Chiou

**Affiliations:** 1 Division of Nephrology, Department of Internal Medicine, Dalin Tzu Chi Hospital, Buddhist Tzu Chi Medical Foundation, Chiayi 622, Taiwan; 2 School of Medicine, Tzu Chi University, Hualien 970, Taiwan; 3 Department of Radiation Oncology, Dalin Tzu Chi Hospital, Buddhist Tzu Chi Medical Foundation, Chiayi 622, Taiwan

We thank Li and colleagues for their insightful comments [1] on our recent work [2] regarding the association of acid suppression therapy with renal and survival outcomes in patients with chronic kidney disease (CKD). In response to their suggestions, we include here two important comorbidities (cerebrovascular disease and malignancy) into the multivariate analysis, which was considered as the leading causes of death among non-dialysis-dependent CKD patients followed in a large healthcare system [3]. We also considered proton pump inhibitor (PPI)–clopidogrel drug–drug interaction in the multivariate analysis because the use of concomitant PPI, which can inhibit cytochrome P450, may attenuate the effectiveness of clopidogrel and increase potential for adverse cardiovascular events [4].

Table 1 shows the percentage of cerebrovascular disease, malignancy, and clopidogrel. Table 2 shows sensitivity analysis when adjusting cerebrovascular disease, malignancy, and PPI–clopidogrel interaction in the original multivariate regression model. The association between H2RA and lower risks of ESRD and overall mortality, as well as between PPI and higher overall mortality, remained significantly consistent. We further compared the risk magnitude of overall mortality across different regression models in the PPI cohort (Table 3) and found the hazard magnitude remained consistent and seemed to be independent of PPI–clopidogrel interaction. This finding was consistent with previous two meta-analyses [5,6] of patients receiving clopidogrel, indicating that concomitant PPI use did not influence overall mortality. Another population-based cohort study in Denmark [4] of 13,001 patients with coronary stent implantation reported that PPI use did not modify the protective effect of clopidogrel, despite the production of a statistically non-significant interaction effect, and concomitant use was not associated with major adverse cardiovascular events. Moreover, two cohort studies [7,8] investigating risk of death among PPI users also did not yet consider PPI–clopidogrel interaction in the analysis. Nevertheless, caution is still warranted when using PPI and clopidogrel concomitantly.

## Figures and Tables

**Table 1:** Adding two comorbidities and one confounding drug in addition to baseline characteristics shown in the original Table 1 ^†^.

	Overall CKD Patients (*n* = 102,802)				Propensity-Matched CKD Patients (*n* = 13,083)			
	PPI Cohort	H2RA Cohort	Control		PPI Cohort	H2RA Cohort	Control	
Variables	(*n* = 7121) N (%)	(*n* = 48,609) N (%)	(*n* = 47,072) N (%)	*p*-value	(*n* = 4361) N (%)	(*n* = 4361) N (%)	(*n* = 4361) N (%)	*p*-value
Comorbidities								
Cerebrovascular disease	708 (9.9)	1957 (4)	2198 (4.7)	<0.0001	269 (6.2)	236 (5.4)	234 (5.4)	0.19
Malignancy	793 (11.1)	4277 (8.8)	3748 (8.0)	<0.0001	438 (10)	291 (6.7)	332 (7/6)	<0.0001
Confounding drugs								
Clopidogrel	985 (13.8)	2655 (5.5)	1349 (2.9)	<0.0001	433 (9.9)	256 (5.9)	164 (3.8)	<0.0001

Abbreviations: CKD, chronic kidney disease; PPI, proton pump inhibitor; H2RA, H_2_-receptor antagonist; NSAID, nonsteroid anti-inflammatory drug; ACEI/ARB, angiotensin-converting enzyme inhibitor/angiotensin receptor blocker. ^†^ Reference 2: J Clin Med, 2022, 11:5612.

**Table 2:** Sensitivity analysis: Adjusted hazard ratio (aHR) for end-stage renal disease (ESRD) and overall mortality in three cohorts.

Outcome	aHR	95% Confidence Interval	*p*-Value
ESRD *			
Control (*n* = 4361)	1.00	Reference	
PPI cohort (*n* = 4361)	1.16	0.92–1.47	0.22
H2RA cohort (*n* = 4361)	0.40	0.30–0.53	<0.0001
Overall mortality ^#^			
Control (*n* = 4361)	1.00	Reference	
PPI cohort (*n* = 4361)	1.83	1.64–2.03	<0.0001
H2RA cohort (*n* = 4361)	0.64	0.57–0.72	<0.0001

Abbreviations: the same as Table 1. * Adjusted for all covariates (age per year, sex, five original comorbidities [diabetes, coronary heart disease, hypertension, acid peptic disease, and chronic liver disease], two new comorbidities [cerebrovascular disease and malignancy], number of medical visits, NSAID, ACEI/ARB, and PPIxclopidogrel interaction term) and competing mortality. ^#^ Adjusted for all covariates (age per year, sex, five original comorbidities [diabetes, coronary heart disease, hypertension, acid peptic disease, and chronic liver disease], two new comorbidities [cerebrovascular disease and malignancy], number of medical visits, NSAID, ACEI/ARB, and clopidogrelxPPI interaction term).

**Table 3:** Compare the risk magnitude of overall mortality across different regression models in the PPI cohort.

	PPI Cohort		
	aHR	95% Confidence Interval	*p*-Value
Model 1: original covariates (age per year, sex, five original comorbidities, number of medical visits, NSAID, and ACEI/ARB)	1.83	1.65–2.03	<0.0001
Model 2: model 1 + adding two comorbidities (cerebrovascular disease, malignancy)	1.81	1.63–2.01	<0.0001
Model 3: model 1 + adding PPIxclopidogrel interaction term	1.84	1.65–2.04	<0.0001
Model 4: model 1 + model 2 + model 3	1.83	1.64–2.03	<0.0001
Model 5: model 1 + model 4 + clopidogrel	1.87	1.68–2.08	<0.0001

Abbreviations: the same as Table 1 and Table 2.
